# Association of Oxidative Stress Markers with Cardio-Kidney-Metabolic Parameters and Cardiovascular Disease in Patients with Type 2 Diabetes Mellitus

**DOI:** 10.3390/biom16010042

**Published:** 2025-12-26

**Authors:** Stefanos Roumeliotis, Ioannis E. Neofytou, Athanasios Roumeliotis, Andrej Veljkovic, Milena Cojic, Gordana Kocic

**Affiliations:** 1 Second Department of Nephrology, School of Medicine, AHEPA Hospital, Aristotle University of Thessaloniki, 54636 Thessaloniki, Greece; john_neofytou_@hotmail.com (I.E.N.); aroumeliotis@auth.gr (A.R.); 2 Institute of Biochemistry, Faculty of Medicine, University of Nis, 18108 Nis, Serbia; andrej.veljkovic@medfak.ni.ac.rs (A.V.); gordana.kocic@ffns.ac.rs (G.K.); 3 Primary Health Care Center, Faculty of Medicine, University of Montenegro, 81100 Podgorica, Montenegro; milenac@ucg.ac.me

**Keywords:** cardiovascular disease, chronic kidney disease, diabetic kidney disease, inflammation, oxidative stress, myeloperoxidase, nitric oxide

## Abstract

We aimed to investigate the association between oxidative stress (OS), inflammation, and kidney function and the predictive ability of OS for mortality and cardiovascular disease in 143 patients with type 2 diabetes (T2DM) and various degrees of kidney function. At baseline, we assessed catalase, nitrogen oxides (NOx), malondialdehyde (MDA), advanced oxidation products (AOPPs), myeloperoxidase (MPO)], kidney function, and C-reactive protein (CRP). All patients were followed for 57 months, with the combined primary outcome of death/cardiovascular (CV) event, whichever occurred first. NOx was an independent predictor of estimated glomerular filtration rate (B = −0.097, *p* = 0.006), and MPO was correlated with glycated hemoglobin (r = 0.17, *p* = 0.046), CRP (r = −0.18, *p* = 0.032), and serum albumin (r = 0.2, *p* = 0.011, Spearman’s rho). During the follow-up, 24 composite events were documented. Kaplan–Meier curves showed that smoking (*p* = 0.029), serum albumin (*p* = 0.014), and MPO (*p* = 0.024, log-rank test) were associated with the outcome. In multivariate Cox regression models, smoking and MPO were independent predictors of the composite outcome (hazard ratio—HR = 2.8, *p* = 0.004, 955 confidence interval—CI 1.05–7.5 and HR = 0.99, *p* = 0.015, 95% CI: 0.98–1.00, respectively), after adjustment for several cofactors. OS might be associated with CV disease in T2DM.

## 1. Introduction

The prevalence and incidence of type 2 diabetes mellitus (T2DM) are rising dramatically worldwide. Specifically, over the past thirty years, the age-standardized prevalence and incidence rate of T2DM worldwide rose from 3.2% to 6.1% (over 90% increase) and from 117 to 183/100,000 population, respectively [1,2]. T2DM prevalence has rapidly increased in low- and middle-income territories and is projected to further increase by 61.2% in 2050, affecting approximately 1.27 billion subjects worldwide [1]. Although during the past twenty years, there has been significant progress regarding the early identification and successful management of several T2DM-derived complications (including peripheral arterial disease, cardiovascular disease [CVD], and retinopathy), until today, the number of diabetic kidney disease (DKD) cases has been continuously rising, reaching pandemic proportions [3]. The main cause of death in these patients is CVD, resulting from accelerated atherosclerosis; patients at early stages of CKD represent 5–7% of the general population and are more likely during their lifetime to suffer a fatal cardiovascular (CV) event than progress to CKD 5D stage and require dialysis. Moreover, CKD contributes to a 41.5% increase in death rate, since it has climbed from 19th to the 12th leading cause of death globally from 2013–2017 and is projected to reach the 5th cause of death by 2040 [4]. This heavy burden that patients with T2DM and CKD carry cannot be solely attributed to traditional CV risk factors, including smoking, obesity, lipidemia, and hypertension, but is more likely to be due to a combination of traditional and novel, uremia, and/or glycemia-related risk factors. Among these, inflammation and oxidative stress (OS) have emerged during the past twenty years as significant risk factors for atherosclerosis, CV events, and death. However, although there is a growing body of evidence suggesting an important role of OS in the pathogenesis and progression of CVD in T2DM and CKD, these data are mainly derived from experimental studies or small clinical studies with surrogate endpoints [5]. Moreover, there are numerous markers of OS (pro-oxidants and anti-oxidants), which remains a major drawback for not implementing the measurement of OS in everyday clinical practice. Malondialdehyde (MDA) is a highly toxic byproduct of lipid peroxidation status, which might reflect early atherogenesis. MDA in diabetes, prediabetes, and CKD impairs endothelial function and promotes vascular calcification and/or stiffens by several pathways [6,7]. Nitric oxide metabolites (NOx) play a major role in kidney function by several molecular pathways; they control overall renal blood flow and glomerular filtration, regulate salt and water excretion, modulate renin secretion, and maintain both renal and systemic arterial blood pressure. In T2DM and CKD, both the generation and functions of NOx are severely decreased, leading to endothelial dysfunction, the hallmark of atherosclerosis [8,9]. Myeloperoxidase (MPO) is a glycosylated, multifunctional, heme-containing leukocyte enzyme with a wide spectrum of actions and functions both in health and disease. MPO is generated by white blood cells during inflammatory conditions and is essential for the neutrophils to kill the attacking microorganisms. MPO might act as a pro-oxidant by generating free radicals (such as hypochlorous acid) and acts as both an anti- and pro-inflammatory mediator [10]. Through its role in immunity, OS, and inflammation, MPO is involved in the pathogenesis and progression of atherosclerosis, since it promotes the oxidative modification of lipoproteins to highly atherogenic molecules. Dysregulated circulating MPO leads to the release of free radicals and oxidants that might directly cause tissue and organ damage that promotes atherogenesis during acute inflammation; therefore, MPO might be a “mechanistic link” connecting inflammation, OS, and the onset or progression of CVD. Although the exact mechanisms underlying these associations remain unknown, several clinical studies have reported that MPO might be a biomarker of CVD in various populations, including patients with established heart failure, acute coronary syndrome, and hypertension. In patients who presented with chest pain at the emergency department, MPO predicted acute myocardial infarction or death even in cases of negative troponin [11]. Catalase is one of the most powerful endogenous antioxidants, which catalyzes the degradation of reactive oxygen species (hydrogen peroxide, specifically to hydrogen and water) and thus protects the cells and tissues from oxidative damage. This enzyme is characterized by a very high turnover rate; even one molecule is able to scavenge millions of hydrogen peroxide molecules [12]. Deficiency of catalase leads to enhanced OS, which triggers the onset of diabetes and/or CKD [13].

Therefore, the possible clinical utility of a variety of OS biomarkers (including markers of protein/lipid oxidation and antioxidants) in T2DM and CKD, the associations of these markers with cardio-kidney-metabolic parameters, and the possible prognostic value for CVD and mortality remain under investigation.

## 2. Materials and Methods

The study was conducted as a prospective observational cohort study in accordance with the standards of the Declaration of Helsinki and the guidelines of Good Clinical Practice [14,15]. The study protocol was approved by the Ethics Committee of the Health Center Podgorica as well as the Ethics Committee of the Faculty of Medicine in Niš (ID number 05/01-E.K.-5989/1). All participants provided written informed consent prior to participation.

We enrolled our study population at baseline and conducted a randomized, controlled trial and 6-month follow-up investigating the role of vitamin D in diabetic patients on metformin therapy. After the end of this study, we stopped any intervention, and we performed a new, separate observational and prospective follow-up study for another 51 months (57 in total) to explore the possible predictive ability of OS markers on mortality and CV events. The present work aims to provide additional insights into the natural long-term of CVD and mortality in this population. All analyses utilize the cohort originally recruited by the researchers and pertain to the same database as described in the previously published investigations [16,17,18].

The original study lasted for six months (from June to December 2018) and was conducted at the Health Center Podgorica in Montenegro. It included male and female patients over the age of 30 with a diagnosis of type 2 diabetes mellitus (T2DM) established according to the criteria of the American Diabetes Association [19] and who were being treated with lifestyle and dietary modifications along with metformin as the only medication. Diagnosis of hypertension and CKD was done according to official guidelines of the European Society of Cardiology (ESC) and the Kidney Disease: Improving Global Outcomes (KDIGO) organization [20,21]. The exclusion criteria were designed to create a homogeneous study population and minimize confounding factors that could significantly affect the results and reduce validity.

Exclusion criteria were as follows:

- Alcoholism;
- Acute inflammatory conditions;
- BMI ≥ 40 kg/m^2^;
- Chronic autoimmune, degenerative, or inflammatory conditions;
- CKD stages 4–5;
- Dementia;
- Gastrointestinal (malabsorption) disorders;
- Liver cirrhosis;
- Malignant disease;
- Medications affecting vitamin D metabolism;
- Pregnancy and breastfeeding;
- Previous use of vitamin D supplementation;
- Urolithiasis;
- Use of other oral antidiabetic drugs (except metformin), with or without insulin therapy.

**Figure 1:**
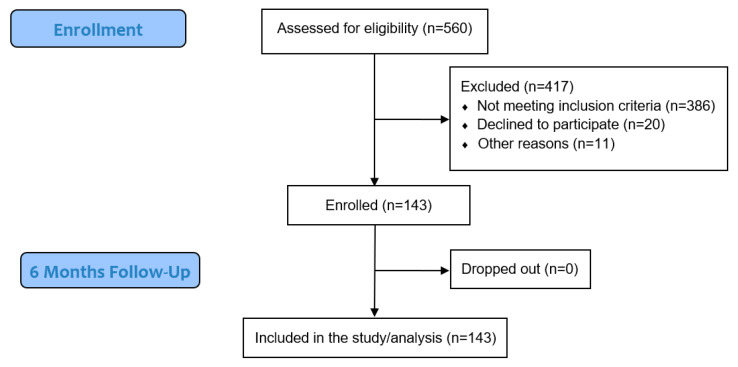
Patient flowchart.

Of a total of 560 patients whose electronic health records were thoroughly reviewed, 174 met all inclusion criteria. Finally, a total of 143 participants agreed and were enrolled in the study (Figure 1).

After enrollment, all patients were followed for an additional 51 months (57 months in total), with the endpoint being the composite outcome of death and/or CV events, whichever occurred first. All enrolled patients completed the follow-up study.

### 2.1. Measurements and Laboratory Analyses

All patients underwent clinical examination and detailed laboratory evaluation at the beginning of the study. The clinical examination included anthropometric measurements and blood pressure (BP) assessment.

#### 2.1.1. Anthropometric Measurements

All anthropometric measurements were performed in the morning and included the determination of body height (BH), body weight (BW), and waist circumference. To ensure accuracy and reliability of the measurements, appropriate conditions and standardized procedures were followed.

Body weight was measured using a calibrated and standardized decimal scale with a possible error of ±100 g. The scale was always placed on a firm surface. Participants were measured after removing outer garments (e.g., jackets, blazers) and footwear. They were asked to step once onto the center of the scale platform for even weight distribution. Body weight was recorded in kilograms.

Body height was measured using a stadiometer (Charder HM200P, Charder Electronic Co., Ltd., Taichung City, Taiwan) mounted vertically against a wall. Participants were asked to remove footwear and stand with their back against the measuring ruler. The back of the head, shoulders, buttocks, calves, and heels had to touch the vertical surface, with heels together and toes slightly apart. Participants were instructed to look straight ahead at a fixed point on the opposite wall. The headpiece of the stadiometer was positioned flat against the hair. Height was recorded to the nearest millimeter/centimeter.

Body mass index (BMI) was calculated using the formula: BMI = BW/BH^2^ (kg/m^2^) [22]. Systolic (SBP) and diastolic blood pressure (DBP) values were measured using automated upper-arm BP monitors (Microlife BP A150-30 AFIB, Microlife, WidnauSwitzerland), which had been previously validated for accuracy.

Before each measurement, participants sat quietly with their back supported and left arm resting so that the brachial artery was at heart level, with the palm facing upward. The upper arm was bare to avoid compression, and the cuff (12 × 15 cm) was placed to cover two-thirds of the arm’s length. After one minute, a second measurement was taken. The final SBP and DBP values were the means of two consecutive readings.

#### 2.1.2. Laboratory Analyses

Blood samples were collected via venipuncture from the cubital vein in the fasting state between 7:00 and 9:00 a.m. after 12 h of overnight fasting. After coagulation, all samples were centrifuged at 2000–3000 rpm to obtain serum. Standard biochemical parameters were analyzed on the same day in the Laboratory Diagnostic Center of the Health Center Podgorica. Serum samples for oxidative stress (OS) markers, analyzed at the Institute of Biochemistry, Faculty of Medicine in Niš, were aliquoted and stored at −80 °C. All assays included daily quality control materials with inter-assay and intra-assay coefficients of variation < 10%.

Serum levels of glucose, urea, creatinine, total cholesterol, low-density lipoprotein (LDL), high-density lipoprotein (HDL), triglycerides (TGs), aspartate aminotransferase (AST), alanine aminotransferase (ALT), and albumin were measured using standard enzymatic procedures (Roche Cobas 6000 c501, Mannheim, Germany). C-reactive protein (CRP) was measured using an immunoturbidimetric method (Roche Cobas 6000 c501, Mannheim, Germany). Hemoglobin A1C (HbA1c) was measured in whole blood samples using the immunoturbidimetric method with K2EDTA anticoagulant (Roche Cobas 6000 c501, Mannheim, Germany). Insulin and vitamin D [25(OH)D3] serum levels were measured by electrochemiluminescence immunoassay using commercial Roche test kits on the Cobas 6000/e601 analyzer (Roche Diagnostics, Mannheim, Germany). Insulin resistance (IR) was assessed using the HOMA–IR (homeostasis model assessment of insulin resistance) formula [23]:

HOMA–IR = glucose (mmol/L) × insulin (μIU/L)/22.5.

The estimated glomerular filtration rate (eGFR) was calculated using the CKD-EPI (2009) equation [24].

##### Oxidative Stress Parameters

Lipid peroxidation was assessed by measuring plasma MDA levels via spectrophotometry, based on the reaction of MDA with thiobarbituric acid (TBA), forming a colored MDA–TBA complex [25]. MDA concentration was expressed in μM/L.

Protein oxidative modification was assessed by measuring plasma advanced oxidation protein product (AOPP) levels spectrophotometrically and expressed as chloramine T equivalents. Plasma samples were diluted 1:10 prior to analysis [26].

Serum MPO activity was measured using a commercial ELISA kit with 96-well microplates pre-coated with biotinylated antibodies (Hycult, Plymouth Meeting, PA). The sandwich assay involved reaction with a detector antibody and a color-producing substrate. MPO enzyme concentration was expressed in ng/L [27].

Serum xanthine oxidase (XO) activity was measured spectrophotometrically by the release of uric acid from xanthine as the substrate [28], with slight modifications of serum assay conditions [29]. XO activity was expressed in U/L.

Serum concentrations of nitrites and nitrates (collectively NOx) were used as indicators of NO production. Nitrate was reduced to nitrite using cadmium-coated copper, and nitrite concentration was determined by colorimetric detection using the Griess reaction [30].

Catalase activity, an antioxidant enzyme, was measured spectrophotometrically using H_2_O_2_ as the substrate, which forms a stable yellow complex with molybdate salts. A decrease in absorbance reflects catalase activity, expressed in kat/L [31].

### 2.2. Statistics

For all statistical analyses, we used the IBM Statistical Package for Social Sciences (SPSS 18.0 for Windows, IBM, Chicago, IL, USA) and R Studio (2025.09.1 Build 401). We checked our data with Kolmogorov–Smirnov test (only HDL-c follows normal distribution, *p* > 0.05). Normally distributed continuous variables are presented as mean ± standard deviation, while non-normally distributed variables are presented as median combined with range (minimum to maximum value). The categorical variables are presented as frequencies and percentages (*n*, %). Differences among two groups were calculated using the Mann–Whitney test for continuous variables or Kruskal–Wallis test for examining more than two groups. Exact Fisher’s test or chi-square test was applied in dichotomous variables. Probability values of *p* < 0.05 (two-tailed) were considered statistically significant [32].

The sample size was based on the feasibility of enrolling eligible participants. A priori power analysis was conducted according to the main study objectives. However, the inclusion of *n* = 143 participants with 23 CV events offers about 80% statistical power (α = 0.05, two-tailed) with the ability to detect hazard ratios (HR) ≥ 1.8 in Cox proportional hazards models [33], which is consistent with effect sizes reported for OS biomarkers in DKD cohorts [34,35,36].

In the first step of our analysis, descriptive statistics were applied to patients’ characteristics and laboratory values. Then, we classified our patients into four different stages of CKD (no CKD and stages 1, 2, and 3) and compared their characteristics. Then, bivariate associations between various OS markers and other variables were examined with Spearman’s correlation coefficient to explore the possible role of redox biology in diabetes and CKD [37]. To investigate the possible predictors of OS status, we conducted a multiple regression analysis (forward, stepwise) with OS markers as the dependent variables in the presence of possible predictors, as determined in the correlation bivariate analyses (variables with *p* < 0.10 in bivariate analysis were entered into stepwise multiple regression, entry criterion *p* < 0.05, removal criterion *p* > 0.10).

Finally, survival analysis was conducted. Due to the limited follow-up duration, event rates (deaths, CV events) could be relatively low, reducing statistical power for multivariable analyses. Therefore, we created a composite cardiovascular endpoint of death and/or CV event, whichever occurred first. We used the Kaplan–Meier method and log-rank tests to compare survival curves. Univariate and multivariate survival analyses were performed using Cox proportional hazard analysis to evaluate adjusted HRs and 95% confidence intervals (CI). The models for all-cause/CV mortality and morbidity were adjusted for well-established confounding factors that were associated with these outcomes in the univariate analysis, including smoking, BMI, and serum albumin.

## 3. Results

In the study cohort, a total of 143 diabetic patients treated with metformin were included in the analysis. Of the patients, 75 were female (52.4%), 12 had a history of CVD (8.4%), 53 had hypertension (37.1%), 121 had CKD stages 1–3 (84.6%), and they reported a 7.1-year median duration of type 2 diabetes mellitus (T2DM). In terms of diabetes control, patients had medians of fasting glucose (7.85 mmol/L), HbA1c (6.74%), and HOMA-IR (3.8). The clinical, anthropometric, hematologic, and biochemical baseline characteristics of the patients are shown in Table 1.

Then, the patients were categorized according to their kidney function status (eGFR) into non-CKD patients (15.4%) and stages 1 (16.8%), 2 (58.7%), and 3 (9.1%) (see Table 2).

**Figure 2:**
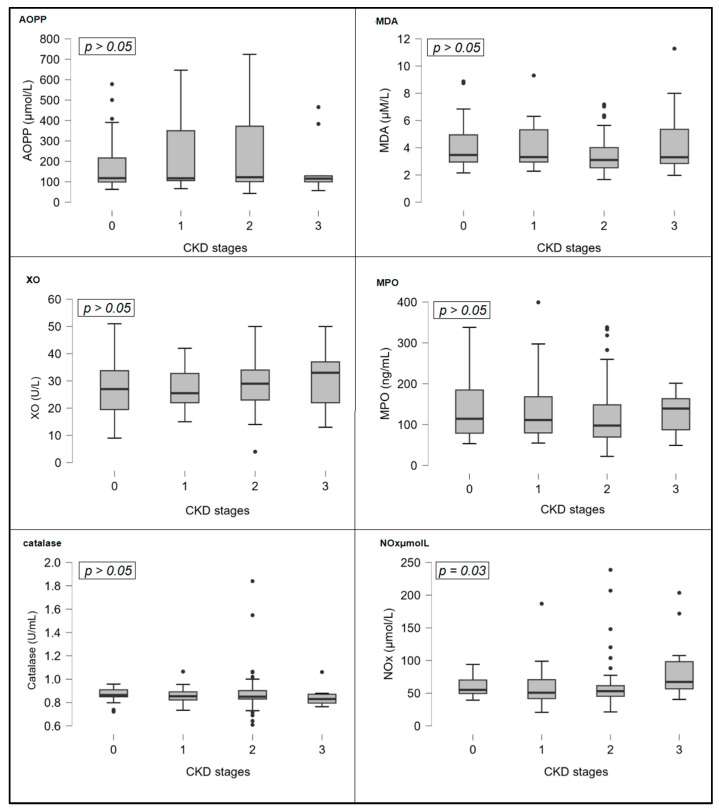
Bar charts of oxidative stress markers concentrations across CKD stages. Abbreviations: AOPP, advanced oxidation protein products; CKD, chronic kidney disease; MDA, malondialdehyde; MPO, myeloperoxidase; NOx, nitric oxide metabolites; XO, xanthine oxidase.

In addition, various OS marker levels were compared along CKD stages. OS markers that represent deranged redox biology (AOPP, MDA, MPO, NOx, and XO) showed a gradual increase following CKD function decline. However, NOx values were found to significantly increase as CKD progressed. (Kruskal–Wallis test, *p* = 0.03). On the other hand, catalase, an antioxidant enzyme, decreased as CKD advanced, but this was not significant (*p* = 0.23). Figure 2 shows bar charts of OS markers across CKD stages.

### 3.1. Correlations and Comparisons Between Groups

Exploration of various relationships between patients’ characteristics and OS markers revealed some statistically significant findings (analyzed with Spearman’s rho test, see Table 3). MPO levels were positively associated with HbA1c (r = 0.167, *p* = 0.04) and serum albumin (r = 0.211, *p* = 0.01) and inversely correlated with CRP (r = −0.180, *p* = 0.032). NOx levels were positively associated with MDA (r = 0.168, *p* = 0.04), serum albumin (r = 0.189, *p* = 0.024), and creatinine (r = 0.173, *p* = 0.038) and inversely correlated with AOPP (r = −0.333, *p* < 0.001). MDA levels were positively associated with HOMA-IR (r = 0.190, *p* = 0.023), total cholesterol (r = 0.220, *p* = 0.008), and triglycerides (r = 0.230, *p* = 0.006) and inversely correlated with AOPP (r = −0.255, *p* = 0.002). Catalase enzyme levels were positively associated with eGFR (r = 0.187, *p* = 0.026), total cholesterol (r = 0.196, *p* = 0.019), LDL (r = 0.188, *p* = 0.024), and triglycerides (r = 0.230, *p* < 0.006). AOPP levels were positively associated with glucose (r = 0.165, *p* = 0.049) and inversely correlated with SBP (r = −0.194, *p* = 0.020), serum albumin (r = −0.227, *p* = 0.006), and NOx and MDA, as stated before. XO levels were positively associated with catalase (r = 0.160, *p* = 0.049). Finally, vitamin D levels were positively associated with diabetes duration (r = 0.203, *p* = 0.015) and inversely correlated with MPO (r = −0.164, *p* = 0.05), triglycerides (r = −0.268, *p* = 0.001), and DBP (r = −0.170, *p* = 0.043).

Further analysis demonstrated that patients with a history of CV disease had significantly lower levels of HbA1c (6.1 vs. 6.6%, *p* = 0.04) and worse renal function (eGFR: 74 vs. 84 mL/min/1.73 m^2^, *p* = 0.01). Healthy patients compared to CKD patients were younger (age: 62 vs. 67, *p* = 0.01) and had higher levels of catalase (0.86 vs. 0.83 U/mL, *p* = 0.02) and lower levels of NOx (52.9 vs. 67.1 μmol/L, *p* < 0.001). Patients were dichotomized between upper median *(n* = 71) and lower median values (*n* = 72) of MPO (median = 103.7 ng/mL, 22.2–399.3). Patients with higher concentrations of MPO showed higher levels of albumin (49 vs. 47.6), lower levels of CRP (1.18 vs. 1.79, *p* = 0.04), and lower levels of catalase (0.85 vs. 0.87, *p* = 0.04) compared to the group with low MPO concentration. Hypoalbuminemia was lower in patients with MPO above the median (38.8% vs. 64.7%, chi-square test, *p* = 0.02). Finally, males, compared to females, had significantly lower levels of vitamin D, (47.7 vs. 63.44 ng/mL, *p* < 0.001), creatinine (72 vs. 90 μmol/L, *p* < 0.001), and albumin (47.45 vs. 49 g/L, *p* < 0.001) (Table 4).

eGFR was correlated with age (*p* < 0.0001, r = −0.34), total and LDL cholesterol (*p* = 0.002, r = 0.25 and *p* = 0.019, r = 0.20), respectively, and catalase (*p* = 0.026, r = 0.19), Spearman’s rho correlation, whereas CKD stages were associated with gender (*p* = 0.05), chi-square test, HbA1c (*p* = 0.04) and NOx (*p* = 0.03), Kruskal–Wallis test. The distribution of female gender according to kidney function was 6/22 (27%) in patients with normal kidney function, 14/24 (58%) in CKD stage 1, 46/84 (54.7%) in CKD stage 2, and 9/13 (69%) in CKD stage 3. Table 5 shows the regression analysis with the dependent variable eGFR and independent variables, which were all those that were associated with either creatinine and/or eGFR and/or CKD stages (age, gender, total/LDL/HDL cholesterol, catalase, history of CVD, serum albumin, HbA1c, NOx). After adjustment for cofactors that were excluded from the multiple regression models (gender, catalase, HDL/LDL cholesterol, albumin, and HbA1c), NOx remained a strong, independent factor predicting eGFR.

Forward stepwise multiple regression analysis was performed to identify predictors of MPO levels. Variables considered for entry included CRP, albumin, HbA1c, and catalase as indicated by the previous results. CRP and catalase failed to show association with MPO and were excluded from the final model (Model 2). Thus, after adjustment for these factors, only serum albumin (B = 7.5, *p* = 0.005, 95% CI 2.25–12.75) and HbA1c (B = 14.6, *p* = 0.019, 95% CI 2.39–26.8) were the sole independent predictors of MPO levels. Albumin emerged as the strongest predictor (standardized or not) across all models (Table 6).

### 3.2. Survival Analysis

**Figure 3:**
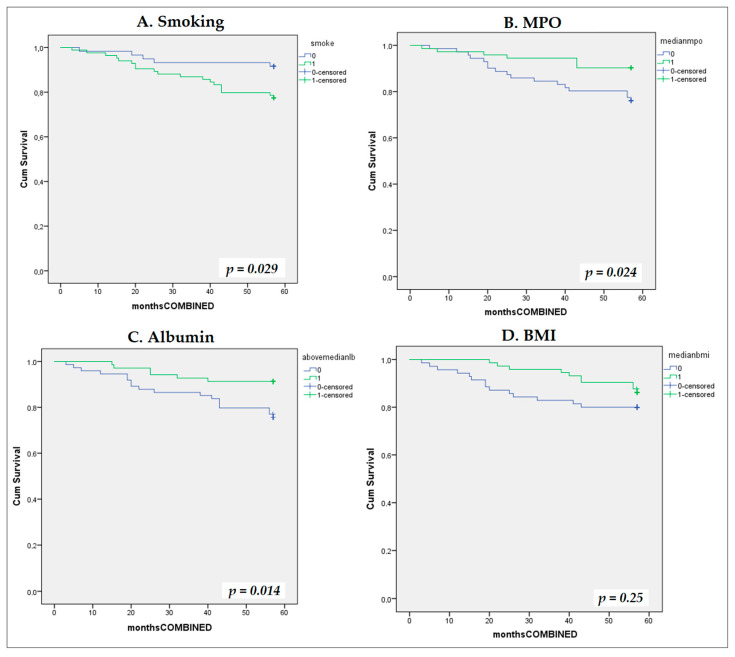
Kaplan–Meier survival curves for composite cardiovascular endpoint stratified by univariate predictors. Kaplan–Meier cumulative incidence curves for the composite cardiovascular endpoint stratified by four univariate predictors: (**A**) smoking status (smokers vs. non-smokers); (**B**) serum myeloperoxidase (MPO) (dichotomized at median: 103.7 ng/mL); (**C**) serum albumin dichotomized at median 48 g/dL; and (**D**) body mass index (BMI) dichotomized at median 29.45 kg/m^2^.

During the median follow-up of 52 months (3–57), we documented eight deaths in total (three deaths from CV causes) and 19 CV events (16 non-fatal). Hence, during the total number of 858 patient-months across 143 patients, 24 composite events occurred (incidence rate: 33.6 per 100 patient-years). Univariate Cox proportional hazard regression models showed that smoking (HR = 2.85, 95% CI 1.06–7.62, *p* = 0.037), MPO (HR = 0.99, 95% CI 0.98–0.99, *p* = 0.014) and albumin as a categorical variable above/below median value (HR = 3.0, 95% CI 1.19–7.60, *p* = 0.02) were associated with the composite outcome, whereas BMI lost its association with the outcome marginally (*p* = 0.07). We inserted MPO into the model as a categorical, dichotomous variable above/below the median value and found that it was associated with the composite outcome (HR = 2.7; 95% CI: 1.10–6.40; *p* = 0.030). Kaplan–Meier curves were performed for each variable: smoking, MPO, BMI, and albumin (Figure 3).

**Figure 4:**
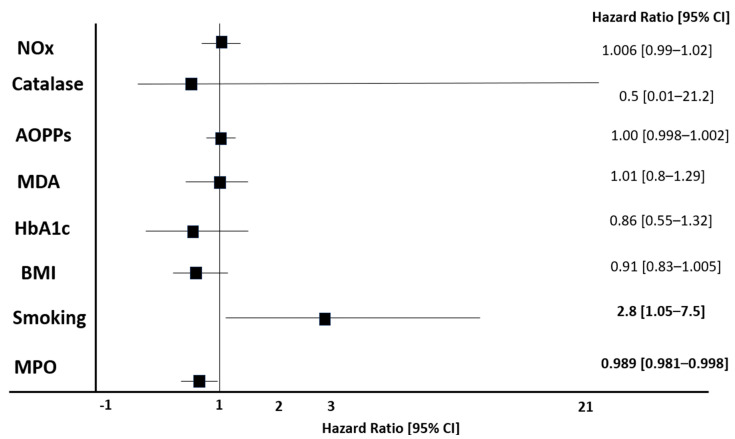
Cox proportional hazards regression analysis using forward stepwise selection for composite cardiovascular endpoint. Model development and variable selection: Forward stepwise selection was employed to identify independent predictors of the composite cardiovascular endpoint. Abbreviations: AOPPs, advanced oxidation protein products; BMI, body mass index; CI, confidence interval; HbA1c, hemoglobin A1c; MDA, malondialdehyde; MPO, myeloperoxidase; NOx, nitrite oxide species.

In multivariate Cox models, we included MPO, BMI, albumin, and smoking as the sole predictors that were identified in the previous survival analysis and showed that smoking and serum MPO were independent predictors of the composite cardiovascular endpoint (Figure 4). The associations remained statistically significant and unchanged after adjustment for other traditional risk factors (Model 3: age, gender, eGFR, CRP, history of CV) and non-traditional, OS-related risk factors (Model 4: HbA1c, Catalase, NOx, AOPPs, MDA), Figure 4.

## 4. Discussion

In a cohort of 143 type 2 diabetes patients treated with metformin, and various degrees of kidney function (ranging from normal to CKD stages 1–3), we found that various markers of OS were associated with kidney function, hypertension, insulin resistance, and lipid/glucose metabolism (Table 2 and Table 3, Figure 1). Moreover, we found that these markers were intra-associated with one another, and MPO was associated with inflammation, and it predicted the primary combined outcome of mortality and/or CV disease (Figure 3 and Figure 4).

In our study, we found a strong correlation between catalase, eGFR, and lipid profile parameters (total/LDL cholesterol, triglycerides), Table 2. Our results are in agreement with previous studies that reported the association between catalase activity and concentration with lipid peroxides and lipoproteins in diabetic patients [38]. In experimental studies, compared to non-diabetic animals, animals with induced diabetes exhibited significantly decreased catalase activity and higher triglycerides, cholesterol levels, and lipid peroxidation status, thus indicating an indirect association between antioxidant activity and lipid metabolism [39]. Moreover, genetic studies show that single-nucleotide polymorphisms of the gene encoding catalase are associated with circulating catalase levels, and lipid biomarkers in patients with type 2 diabetes (effects of rs769217 and rs1001179 polymorphisms of catalase gene on blood catalase, carbohydrate and lipid biomarkers in diabetes mellitus) and reduced risk of CKD [40,41]. In agreement with our results, data from experimental CKD models suggest that eGFR decline is associated with decreased catalase activity [42], whereas small clinical studies have also shown that catalase activity is gradually deteriorating along with CKD progression and worsening kidney function [43].

In our cohort, we found a strong correlation between NOx and kidney function, assessed by serum creatinine and eGFR (Table 3 and Figure 2). Circulating NOx is correlated with flow-mediated dilation and intima-media thickness, thus reflecting the severity of endothelial dysfunction [44]. Moreover, NOx bioavailability and bioactivity are important regulators and mediators of aging, DKD, acute and chronic inflammation, OS, and cardiovascular-kidney-metabolic disorders. In our study, we found that NOx was significantly different among CKD stages; moreover, NOx remained a strong independent predictor of eGFR value, even after adjustment for several traditional cofactors, including gender, LDL cholesterol, and HbA1c (Table 5). Furthermore, NOx was associated with MDA and AOPPs. This is in accordance with previous studies reporting the strong association between NOx bioavailability and OS in diabetes and DKD [45,46]. Our finding that NOx is tightly associated with kidney function is in agreement with experimental studies, where renal production and the expression of NO were significantly affected by DKD and triggered endothelial dysfunction and kidney injury [46,47]. Even in kidney transplant patients, nitric oxide predicted kidney outcomes, including graft function and post-transplantation eGFR [48].

MDA is a highly toxic byproduct of lipid peroxidation status, which might reflect early atherogenesis. This explains our finding that MDA levels were associated with cholesterol and triglycerides (Table 3). In chronic hyperglycemia, insulin resistance and other metabolic changes—including deranged lipid metabolism—trigger the generation of reactive oxygen species (ROS), increased OS, and the overproduction of MDA [49]. We found that circulating MDA levels in diabetics were associated with NOx and HOMA (Table 3). These findings are in accordance with other studies reporting that insulin resistance triggers overgeneration of circulating MDA and causes endothelial dysfunction in obese, prediabetic, and T2DM patients [50,51]. MDA in diabetes, prediabetes, and CKD impairs endothelial function and promotes vascular calcification and/or stiffness by several pathways: it modifies LDL molecules, promotes the formation of foam cells, triggers the peroxidation of cell membrane lipids, and upregulates vascular inflammation and disturbs the immune system. This is why several clinical studies have reported that MDA predicts carotid endothelial atherosclerosis [52] and arterial stiffness [53,54,55].

Free radicals often attack proteins to cause dramatic modifications in their structure and function. The result of this oxidative attack in serum albumin is the formation of AOPPs, which were first discovered in uremic patients [56]. This explains our finding that AOPPs were inversely correlated with circulating albumin (Table 3). We also found a strong association between circulating AOPPs and systolic blood pressure and NOx. OS, especially the oxidation of serum proteins, is implicated in the pathogenesis and progression of hypertension and subsequently endothelial dysfunction. In a small population of 75 ESKD patients undergoing peritoneal dialysis, Xu et al. found that even adjustment for several cofactors, AOPPs were independent predictors of both central diastolic and systolic blood pressure [57], whereas Conti et al. showed that compared to healthy controls, hypertensive patients exhibit significantly higher circulating AOPP levels [58]. Through this and other mechanisms, AOPPs have been implicated in the progression of atherosclerosis and arterial stiffness, conditions characterized by impaired NOx bioavailability [6,7]. MPO mediates bacteria killing by leukocytes, increases the toxicity of leukocyte-formatted free radicals, and therefore is considered to act as a pro-inflammatory cytokine. Despite the fact that the clinical impact of low MPO in humans has not yet been fully elucidated, it has been reported that subjects with MPO deficiency or insufficiency experience a high incidence of chronic inflammation, thus suggesting that low MPO is associated with increased inflammation [59,60]. Moreover, single genetic polymorphism that results in downregulated MPO are prone to develop autoimmune diseases like multiple sclerosis and lupus nephritis, whereas patients with type 2 diabetes typically have significantly reduced MPO activity in their white blood cells [61]. These data are in agreement with our findings that low MPO is associated with increased inflammation status and might be attributed to the fact that in animal models lacking MPO, there is marked development of adaptive immune responses and T-cell-mediated tissue inflammation, possibly by abrogating dendritic cell function [62,63,64,65]. Therefore, decreased MPO might derange the T-cell adaptive immunity, making it easier for inflammation to develop and progress. Inflammation and OS play a pivotal role in the pathogenesis of atherosclerosis. MPO is expressed in atherosclerotic tissues and catalyzes the oxidative alteration of LDL molecules and the formation of foam cells [66] and mediates the oxidation of proteins that have been located in atherosclerotic sites [67]. Taken together, these experimental data support that MPO might mediate inflammation and oxidation of proteins and lipoproteins to trigger the onset of atherosclerosis. In clinical studies, elevated MPO has been shown to be predictive of a new CV event (coronary heart disease) in patients presenting with chest pain [11,68] and in patients with heart failure [69] and/or coronary heart disease [70].

However, experimental in vivo studies have shown that reduced levels of circulating MPO lead to a drastic, exacerbated inflammatory response and cytokine production, acting as an anti-inflammatory mediator [10,71]. We found a strong inverse correlation of MPO with CRP, which might be explained by the aforementioned data (Table 3 and Table 4). Moreover, we found that MPO was a strong independent predictor of mortality or CV event after a 5-year follow-up, in a cohort of 143 type 2 diabetes patients. In our study, low levels of MPO were associated with increased inflammation and onset of CV events (Figure 3 and Figure 4). Although high MPO has been associated with coronary heart disease and CV disease, it might be hypothesized that, similarly to inflammation, MPO at low dosages might act as an atherogenic molecule.

To further investigate the implication of MPO in atherosclerosis, Brennan et al. developed MPO-knockout animal models that were subjected to a hyperlipidemic diet, which induced atherosclerosis, and found that compared to controls, mice with MPO deficiency had 50% more accelerated atherosclerotic lesions in their aorta [72]. When a genetic cross was performed with LDL receptor knockout animal models, the results were practically the same, indicating that reduced MPO levels promoted atherogenesis. Although in our study, we found inter-associations between various markers of OS (AOPPs, MDA, NOx, catalase), MPO was not associated with any of these markers and was negatively correlated with CRP. Experimental studies have reported that MPO might ameliorate the oxidative injury of inflammatory, chemotactic cytokines [73], modify the actions of lymphocytes [74], upregulate proteases that regulate white blood cell infiltration [75], inactivate the deleterious effects of inflammatory molecules [76,77], and abrogate the oxidative turnover of lipoproteins [78]. MPO might also influence the generation of oxidized lipoproteins and subsequently affect macrophage and vascular smooth muscle cell proliferation [76] and regulate the bioavailability and action of NO [79] or NO-generated free radicals [76,80,81]. Finally, the anti-atherogenic effect of MPO might also be attributed to the impaired ability to wipe out factors and molecules with known atherogenic properties, including oxidized LDL cholesterol [82,83]. This is why it is suggested that MPO could be implicated in all stages of atherosclerosis and inflammation and might act either as a pro- or anti-atherogenic and pro- or anti-inflammatory molecule.

Albuminuria (urine albumin to creatinine ratio-UACR) is a key element of DKD resulting from NOx and ROS-mediated glomerulopathy and injury of the podocytes. UACR has been associated with increased OS and inflammation in DKD [84], whereas large, epidemiological studies coherently showed that UACR is a strong, independent factor for mortality and CVD and DKD progression [85]. Although UACR was considered for decades the first, essential step of DKD, recently, it became evident that DKD might exist in two different phenotypes, the albuminuric and the non-albuminuric form [49].

There are certain limitations to our study that should be acknowledged. Firstly, the observational design precludes any causality of the results. Secondly, the sample size could have been bigger and the follow-up period longer, in order for more events to occur. Thirdly, there were many patients who were screened and initially excluded from the study; this could mean that our study may have introduced selection bias. An important limitation and drawback that should definitely be acknowledged is also the fact that we did not have data on UACR, which is a major risk factor for CVD and mortality and is associated with OS. Finally, and most importantly, due to the complex pathophysiology of T2DM, where all conditions are somehow interrelated, our associations could be attributed to epiphenomena. For example, the association of MPO with mortality and CV events could be attributed to the tight correlation between MPO and CRP. Therefore, our findings should be interpreted with caution. However, although OS has been in the center of scientific research in diabetes and CKD during the past two decades, clinical studies with hard outcomes are very limited, and therefore, the clinical utility of OS biomarkers in these conditions remains undefined. Our study indicates and hypothesizes that OS, along with inflammation, is implicated in diabetes and CKD. Large, well-designed, prospective studies with large sample sizes, long follow-up periods, and clinical hard outcomes are needed in order to draw definite conclusions.

## 5. Conclusions

In a cohort of 143 patients with documented T2DM treated with metformin and various degrees of kidney function, we found associations between biomarkers of OS (both oxidants and antioxidants) and kidney function, insulin resistance, and endothelial dysfunction. Specifically, as eGFR declined and CKD progressed, catalase was decreased, and NOx was increased. Moreover, low circulating MPO was associated with increased inflammation and was an independent predictor of the composite outcome of mortality and/or CV event after a nearly 6-year follow-up period. Our study supports that markers of OS might be implicated in the pathogenesis of CV disease in patients with diabetes and/or CKD.

## Figures and Tables

**Table 1:** Clinical, anthropometric, and biochemical characteristics of the patients.

	All Patients
	(*n* = 143)
Age (years)	61.5 (33, 82)
Gender, Male (%)	47.6 (68)
Smoking (%)	37 (53)
History of CV disease (%)	8.4 (12)
CKD stages 1–3 (%)	84.6 (121)
Hypertension (%)	37.1 (53)
Duration of T2DM (years)	7.1 (1, 14)
BMI (kg/m^2^)	29.45 (17.3, 46.6)
SBP (mm Hg)	132.5 (87, 198)
DBP (mm Hg)	80 (53, 117)
Glucose (mmol/L)	7.85 (5.3, 21)
Vitamin D (nmol/L)	56.1 (19.1, 127.6)
Creatinine (μmol/L)	82 (47, 157)
eGFR (mL/min/1.73 m^2^)	82.2 (38, 116)
HOMA-IR	3.8 (0, 25)
Albumin (g/L)	48 (44, 55)
Total cholesterol (mmol/L)	5.28 (3.0, 10.13)
LDL-cholesterol (mmol/L)	3.26 (1.67, 9.92)
HDL-cholesterol (mmol/L)	1.31 ± 0.3
Triglycerides (mmol/L)	1.69 (0.46, 26)
HbA1c (%)	6.74 (4.9, 10)
CRP (mg/L)	1.61 (0.3, 22)

Results for continuous variables are presented as median (range) or mean (S.D.). Results from categorical variables are presented as frequencies and percentages (n, %). Abbreviations: CV, cardiovascular; CKD, chronic kidney disease; T2DM, type 2 diabetes mellitus; BMI, body mass index; SBP, systolic blood pressure; DBP, diastolic blood pressure; eGFR, estimated glomerular filtration rate; HOMA-IR, homeostatic model assessment for insulin resistance; LDL, low-density lipoprotein; HDL, high-density lipoprotein; HbA1c, glycated hemoglobin A1c; CRP, C-reactive protein.

**Table 2:** Comparison between patients’ characteristics stratified by CKD stage.

Characteristic	Non-CKD	Stage 1	Stage 2	Stage 3	*p*
*N*	22	24	84	13	
Age (years)	55.50 [45, 72]	61.5 [33, 74]	63 [42, 78]	67 [50, 82]	0.001
Gender, Male (%)	16 (72.2)	10 (41.7)	38 (45.2)	4 (30.8)	0.050
Smoking (%)	13 (59.1)	15 (62.5)	48 (57.1)	8 (61.5)	0.978
Hypertension (%)	10 (45)	7 (29.1)	28 (33.3)	8 (61.5)	0.161
Combined CV event (%)	4 (18.2)	5 (20.8)	12 (14.3)	3 (23.1)	0.687
Duration of T2DM (years)	6 [1, 12]	6 [1, 13.7]	7.5 [1, 12.5]	7 [4, 12]	0.651
BMI (kg/m^2^)	29.07 [21.82, 35.83]	28.25 [17.73, 37.58]	29.68 [17.30, 46.60]	30.66 [24.58, 34.52]	0.569
SBP (mm Hg)	132.25 [109, 170.5]	128.5 [87, 198.5]	133.75 [90, 185]	141.0 [13, 173.5]	0.077
DBP (mm Hg)	83.5 [67.5, 106.5]	79 [53.5, 110]	79 [60, 117.5]	82.5 [64.5, 100.5]	0.256
Glucose (mmol/L)	7.55 [5.3, 15.5]	8.25 [6.4, 21]	7.75 [5.3, 13.1]	8.00 [5.9, 9.1]	0.295
Vitamin D	44.84 [19.1, 127.6]	51.56 [19.48, 110.8]	58.36 [22.02, 120.1]	53.85 [22.81, 84.49]	0.167
HOMA-IR	3.01 [0.73, 11.82]	3.98 [0.89, 22.56]	3.90 [0.0, 25.04]	4.23 [1.32, 11.41]	0.619
Albumin (g/L)	48.50 [45, 55]	48.36 [46, 52]	48.00 [44, 55]	48.00 [44, 53]	0.774
Total cholesterol (mmol/L)	6.24 [3.50, 10.13]	5.42 [4.06, 7.73]	5.20 [3.09, 9.67]	4.90 [3.79, 6.39]	0.015
LDL-cholesterol (mmol/L)	3.87 [1.67, 7.16]	3.20 [1.98, 9.92]	3.26 [1.36, 6.25]	2.60 [1.99, 4.42]	0.050
HDL-cholesterol (mmol/L)	1.29 [0.86, 2.32]	1.39 [0.80, 2.04]	1.33 [0.78, 2.17]	1.21 [0.70, 1.71]	0.777
Triglycerides (mmol/L)	1.65 [0.71, 26.02]	1.95 [0.66, 9.26]	1.67 [-0.46, 5.40]	1.80 [1.12, 5.36]	0.361
HbA1c (%)	6.45 [5.1, 9.4]	6.98 [6.0, 10.1]	6.47 [4.9, 8.8]	6.50 [5.4, 7.8]	0.040
CRP (mg/L)	1.67 [0.30, 22.07]	1.00 [0.31, 8.68]	1.73 [0.30, 15.46]	1.40 [0.30, 10.54]	0.342
AOPP (µmol/L)	117.90 [63.11, 578.23]	117.17 [66.53, 646.72]	122.54 [43.05, 724.26]	115.45 [57.24, 465.72]	0.573
MDA (μΜ/L)	3.47 [2.15, 8.87]	3.32 [2.28, 9.31]	3.10 [1.67, 7.18]	3.31 [1.97, 11.28]	0.229
MPO (ng/mL)	114.28 [53.44, 338.08]	111.27 [54.90, 399.30]	97.70 [22.24, 338.37]	139.31 [49.10, 201.34]	0.420
NOx (μmol/L)	55.13 [39.36, 94.02]	50.69 [20.69, 186.91]	53.02 [21.36, 238.70]	67.13 [40.47, 203.58]	0.031
Catalase (U/mL)	0.86 [0.72, 0.96]	0.85 [0.73, 1.06]	0.85 [0.61, 1.84]	0.83 [0.76, 1.06]	0.225
Xanthine Oxidase (U/L)	27.0 [9, 59]	25.5 [15, 42]	29 [4, 50]	33 [13, 50]	0.786

Data presented as median (minimum, maximum) for continuous variables and n (%) for categorical variables. Abbreviations: CKD, chronic kidney disease; BMI, body mass index; SBP, systolic blood pressure; DBP, diastolic blood pressure; HOMAIR, homeostatic model assessment of insulin resistance; LDL, low-density lipoprotein; HDL, high-density lipoprotein; HbA1c, glycated hemoglobin; CRP, C-reactive protein; AOPP, advanced oxidation protein products; MDA, malondialdehyde; MPO, myeloperoxidase; NOx, nitric oxide metabolites. *p*-values from Kruskal–Wallis test for continuous variables and Fisher’s exact or chi-squared test for categorical variables.

**Table 3:** Correlations of OS and vitamin D with various parameters.

			*r*		*p*	
MPO	-		HbA1c	0.167	*	0.046
MPO	-		Serum albumin	0.211	*	0.011
MPO	-		CRP	−0.180	*	0.032
NOx	-		MDA	0.168	*	0.045
NOx	-		AOPP	−0.333	***	<0.001
NOx	-		Creatinine	0.173	*	0.038
NOx	-		Serum albumin	0.189	*	0.024
MDA	-		NOx	0.168	*	0.045
MDA	-		AOPP	−0.255	**	0.002
MDA	-		HOMA-IR	0.190	*	0.023
MDA	-		Cholesterol	0.220	**	0.008
MDA	-		Triglycerides	0.320	***	<0.001
Catalase	-		XO	0.165	*	0.049
Catalase	-		eGFR	0.187	*	0.026
Catalase	-		Cholesterol	0.196	*	0.019
Catalase	-		Triglycerides	0.230	**	0.006
Catalase	-		LDL-C	0.188	*	0.024
AOPP	-		NOx	−0.333	***	<0.001
AOPP	-		MDA	−0.255	**	0.002
AOPP	-		Glucose	0.165	*	0.049
AOPP	-		Systolic BP	−0.194	*	0.020
AOPP	-		Serum albumin	−0.227	**	0.006
XO	-		Catalase	0.165	*	0.049
CRP		-	Albumin	−0.3	***	<0.0001
CRP		-	BMI	0.3	***	<0.0001
CRP		-	HOMAIR	0.22	**	0.01
CRP	-	-	HDL cholesterol	−0.25	**	0.002
Vitamin D	-	-	Diabetes duration	0.203	*	0.015
Vitamin D	-	-	Triglycerides	−0.268	**	0.001
Vitamin D		-	Diastolic BP	−0.17	*	0.043

r, Spearman’s rho test correlation; various degrees of statistical significance, * *p*-value < 0.05, ** *p* < 0.01, *** *p* < 0.001; Abbreviations: AOPP, advanced oxidation protein products; BMI, body mass index; catalase, catalase enzyme; cholesterol, total cholesterol; CRP, C-reactive protein; eGFR, estimated glomerular filtration rate; HbA1c, hemoglobin A1c; HOMAIR, homeostatic model assessment of insulin resistance; LDL-C, low-density lipoprotein cholesterol; MDA, malondialdehyde; MPO, myeloperoxidase; NOx, nitric oxide species; systolic BP, systolic blood pressure; diastolic BP, diastolic blood pressure; XO, xanthine oxidase.

**Table 4:** Comparative analysis of clinical and biochemical parameters based on cardiovascular history, glomerular filtration rate, myeloperoxidase levels, and gender.

	No CV History (*n* = 131)	CV History (*n* = 12)		
Variable	Median (Min, Max)	Median (Min, Max)	*p*-Value	Sig.
HbA1c (%)	6.6 (4.9, 10.10)	6.1 (5.4, 7.4)	0.047	*
Creatinine (μmol/L)	80.00 (47.00, 157.00)	92.70 (68.00, 127.00)	0.02	*
GFR (mL/min/1.73 m^2^)	84.00 (38.00, 116.00)	74.00 (42.00, 89.00)	0.01	*
	GFR > 60 mL/min (*n* = 130)	GFR < 60 mL/min (*n* = 13)		
Variable	Median (min, max)	Median (min, max)	*p*-value	Sig.
Age (years)	62.00 (33.00, 78.00)	67.00 (50.00, 82.00)	0.014	*
Catalase (U/mL)	0.86 (0.64, 1.84)	0.83 (0.61, 1.06)	0.023	*
NOx (μmol/L)	52.91 (20.69, 238.70)	67.13 (40.47, 206.91)	0.001	**
	Low MPO (*n* = 71)	High MPO (*n* = 72)		
Variable	Median (min, max)	Median (min, max)	*p*-value	Sig.
CRP (mg/L)	1.79 (0.30, 22.07)	1.18 (0.30, 15.34)	0.047	*
Albumin (g/L)	47.64 (44.00, 54.00)	49.00 (44.00, 55.00)	0.006	**
Catalase (U/mL)	0.87 (0.64, 1.84)	0.85 (0.61, 1.06)	0.04	*
	Females (*n* = 68)	Males (*n* = 75)		
Variable	Median (min, max)	Median (min, max)	*p*-value	Sig.
Vitamin D (ng/mL)	47.70 (19.10, 127.60)	63.44 (29.41, 120.10)	0.0006	***
Creatinine (μmol/L)	72.00 (47.00, 123.00)	90.00 (57.00, 157.00)	0.0000	***
Albumin (g/L)	47.45 (44.00, 53.00)	49.00 (44.00, 55.00)	0.0007	***

Data presented as median (minimum, maximum). Non-parametric tests were conducted using the Mann–Whitney U test. Significance levels: *** *p* < 0.001, ** *p* < 0.01, * *p* < 0.05. Abbreviations: CRP, C-reactive protein (mg/L); CV, cardiovascular; GFR, glomerular filtration rate (mL/min/1.73 m^2^); HbA1c, hemoglobin A1c (%); MPO, myeloperoxidase; max, maximum; min, minimum; NOx, nitrate oxide species.

**Table 5:** Stepwise multiple regression models predicting eGFR.

	B	Std Error	*p*	95% CI for B
Model 1				
Age	−0.65	0.14	<0.0001	−0.94 to −0.37
Model 2				
Age	−0.60	0.13	<0.0001	−0.86 to −0.33
Total Cholesterol	2.67	0.90	0.005	0.89–4.46
History of CVD	−9.4	4.3	0.03	−17.9 to −0.95
NOx	−0.097	0.04	0.006	−0.17 to −0.03

Gender, LDL/HDL cholesterol, catalase, serum albumin, and HbA1c were excluded from Model 2. eGFR, estimated glomerular filtration rate; CI, confidence interval. B (beta) is the regression coefficient estimating the change of eGFR for every unit of increase in each independent variable (age, total cholesterol, history of CVD, NOx), while all other variables remain unchanged.

**Table 6:** Stepwise multiple regression models predicting serum MPO concentrations.

	B	Std Error	*p*	95% CI for B
Model 1				
Serum Albumin	7.79	2.7	0.004	2.46–13.12
Model 2				
Serum Albumin	7.50	2.66	0.005	2.25–12.75
HbA1c	14.6	0.19	0.019	2.4–26.8

CRP and catalase were excluded from Model 2.

## Data Availability

The original contributions presented in this study are included in the article. Further inquiries can be directed to the corresponding author.
